# The elastin peptide VGVAPG increases CD4^+^ T-cell IL-4 production in patients with chronic obstructive pulmonary disease

**DOI:** 10.1186/s12931-020-01609-4

**Published:** 2021-01-13

**Authors:** Flora Lemaire, Sandra Audonnet, Jeanne-Marie Perotin, Pierre Gaudry, Sandra Dury, Julien Ancel, François Lebargy, Frank Antonicelli, Gaëtan Deslée, Richard Le Naour

**Affiliations:** 1 Laboratory of Immunology, EA7509 IRMAIC, University of Reims Champagne-Ardenne (URCA), Reims, France; 2 Flow Cytometry Platform URCACyt, URCA, Reims, France; 3 Department of Pulmonary Medicine, University Hospital of Reims, Reims, France; 4 INSERM U1250, URCA, Reims, France

**Keywords:** COPD, T cells, Elastin peptides, Cytokines, IL-4, Flow cytometry

## Abstract

**Background:**

In chronic obstructive pulmonary disease (COPD), lung-infiltrating inflammatory cells secrete proteases and participate in elastin breakdown and genesis of elastin-derived peptides (EP). In the present study, we hypothesized that the pattern of T lymphocytes cytokine expression may be modulated by EP in COPD patients.

**Methods:**

CD4^+^ and CD8^+^ T-cells, sorted from peripheral blood mononuclear cells (PBMC) collected from COPD patients (n = 29) and controls (n = 13) were cultured with or without EP. Cytokine expression in T-cell phenotypes was analyzed by multicolor flow cytometry, whereas desmosine concentration, a specific marker of elastin degradation, was measured in sera.

**Results:**

Compared with control, the percentage of IL-4 (Th2) producing CD4^+^ T-cells was decreased in COPD patients (35.3 ± 3.4% and 26.3 ± 2.4%, respectively, *p* < 0.05), whereas no significant differences were found with IFN-γ (Th1) and IL-17A (Th17). Among COPD patients, two subpopulations were observed based on the percentage of IL-4 (Th2) producing CD4^+^ T-cells, of which only one expressed high IL-4 levels in association with high levels of desmosine and strong smoking exposure (n = 7). Upon stimulation with VGVAPG, a bioactive EP motif, the percentage of CD4^+^ T cells expressing IL-4 significantly increased in COPD patients (*p* < 0.05), but not in controls. The VGVAPG-induced increase in IL-4 was inhibited in the presence of analogous peptide antagonizing VGVAPG/elastin receptor (S-gal) interactions.

**Conclusions:**

The present study demonstrates that the VGVAPG elastin peptide modulates CD4^+^ T-cells IL-4 production in COPD. Monitoring IL-4 in circulating CD4^+^ T-cells may help to better characterize COPD phenotypes and could open a new pharmacologic opportunity through CD4^+^ T-cells stimulation via the VGVAPG/S-gal receptor in order to favor an anti-inflammatory response in those COPD patients.

## Background

Chronic obstructive pulmonary disease (COPD) is a chronic inflammatory disease characterized by progressive airflow limitation associated with an excessive inflammatory response to inhaled particles, mainly tobacco smoking [1]. COPD pathology includes obstruction of small airways and emphysema that is characterized by the destruction of lung parenchyma resulting in loss of lung elasticity. The inflammatory process in COPD involves inflammatory cells, predominantly macrophages and neutrophils [2], and immune cells such as NK cells [3], NKT cells [4] and T lymphocytes. Data from the literature report the presence of CD4^+^ and CD8^+^ T lymphocytes in both airways and parenchyma in COPD [5–7] as well as relationships between increase in these cell populations and COPD severity [8–10]. Several studies attempted to characterize the pattern of cytokines produced by T lymphocyte during airway remodeling in COPD with controversial results. Indeed, while studies showed predominance of type 1 cytokines expressed by CD4^+^ (T helper (Th) 1) and CD8^+^ T lymphocytes (T cytotoxic (Tc) 1) [5, 8, 11, 12], others described either a Th2/Tc2 [13, 14] or a Th17/Tc17 [12, 15, 16] profile in the airways, peripheral blood, and/or the broncho-alveolar lavages (BAL) of COPD patients.

Studies in human with severe α-1 anti-trypsin deficiency [17] and in animal models exposed to proteases [18, 19] suggest that tissue damage associated with COPD partly results from proteolytic breakdown of extracellular matrix proteins by proteases released from lung inflammatory cells [20]. Some of these proteases promote degradation of pulmonary elastin [21]. Accordingly, increased secretion of desmosine, a specific marker for elastin degradation [22], and elevated levels of soluble elastin peptides (EP) in various biological fluids reflect massive pulmonary elastin breakdown in COPD patients [23, 24].

EP display a wide range of biological activities that are largely mediated through their interaction with a 67-kDa, an external subunit of the elastin receptor, designed as spliced-galactosidase (S-gal) [25]. Noteworthy, we previously showed that EP could modulate the expression of pro-inflammatory cytokines in monocytes from healthy subjects and favour a Th1 cytokine response in lymphocytes [26, 27]. We also previously reported that EP/S-gal interactions impaired neutrophil-dependent cytokine expression in COPD patients [24] and demonstrates that in a murine model of emphysema, EP-S-gal interactions contribute to a Th1 and Th17 proinflammatory T-cell response [28]. Moreover, we observed that exposure of mice to EP elicited hallmark features of emphysema with inflammatory cell accumulation associated with increased matrix metalloproteinases and desmosine expression associated with airway remodelling [29]. Therefore, we wondered whether such a feedback loop linking inflammation, cytokine, proteases and elastine breakdown could also be of involved in COPD.

In this study, we hypothesized that the pattern of cytokine expression of CD4^+^ and CD8^+^ T lymphocytes in patients with COPD could be modulated by EP/T-cells interactions. We demonstrated that EP modulate interleukin (IL)-4 production by CD4^+^ T-cells from peripheral blood from COPD patients, and that activating effect of EP on IL-4 production is mediated by S-gal receptor.

## Methods

### Subjects’ characteristics

Controls and COPD patients were prospectively recruited from the Department of Pulmonary Medicine at the University Hospital of Reims (France) and included in the cohort for Research and Innovation in Chronic Inflammatory Respiratory Diseases (RINNOPARI, NCT02924818). The study was approved by the ethics committee for the protection of human beings involved in biomedical research (CPP Dijon EST I, No. 2016-A00242-49). All subjects gave written informed consent prior to inclusion in the study. Controls (n = 13) were recruited from subjects attending the department of pulmonary medicine for pulmonary function tests (PFT) and were invited to participate in the study if they did not present any acute or chronic respiratory or allergic disease. COPD patients (n = 29) were enrolled in the study on the basis of clinical and functional assessments with a forced expiratory volume in 1−s (FEV_1_)/forced vital capacity (FVC) < 0.7 after bronchodilation. At inclusion, all patients were stable with no acute exacerbation of COPD for 4 weeks. Patients with asthma, allergic disease, tuberculosis, neoplasia, or other chronic respiratory diseases were excluded. Characteristics of the controls and COPD patients including demographic data, medical history, smoking history (non-smokers, ex-smokers, current smokers), respiratory symptoms, PFT, and peripheral blood eosinophil count were collected. A thoracic CT-scan including a quantitative emphysema score was performed in each COPD patients. Blood and *sera* samples were collected from each controls and COPD patients.

### Lymphocyte isolation, culture and treatment

Peripheral blood mononuclear cells (PBMC) were collected from heparinized whole blood using a density gradient medium (Pancoll™, PAN-Biotech, Aidenbach, Germany). Red blood cells were then lysed using RBC Lysis Solution (Miltenyi Biotec, Paris, France) and PBMC washed with X-VIVO 15 serum free medium (Lonza, Verviers, Belgium). CD4^+^ and CD8^+^ T-cells were separately isolated from PBMC by immuno-magnetic depletion using human CD4^+^ T-cell and human CD8^+^ T-cell isolation kits respectively, according to the manufacturer’s instructions (Miltenyi Biotec). T-cell purity and viability were respectively over 95% and 98% as determined by flow cytometry analysis (data not shown). Absence of detectable levels of IFN-γ, IL-4 and IL-17A expressed by unactivated CD4^+^ T-cells or CD8^+^ T-cells (data not shown) led us to culture the two-sorted T-cell subsets with anti-CD3/CD28 coated beads. Thus, isolated CD4^+^ T-cells and CD8^+^ T-cells were cultured in 96-well culture plates (5 × 10^5^ cells/well) and incubated with Dynabeads human T-activator CD3/CD28 (Invitrogen, Illkirch, France) in the presence or absence of the VGVAPG elastin peptide (10 µg/mL, Genepep SA, Saint Jean de Védas, France). After 48 h incubation, stimulated cells were collected, stained and analyzed by flow cytometry to evaluate the levels of IFN-γ, IL-4 and IL-17A cytokine expression. Cell culture supernatants were collected for cytokine quantification by Cytometric Bead Array (CBA) Flex Set technology (BD Biosciences, Le Pont de Claix, France). We previously identified the analogous antagonist peptide PGAIP as an inactive C-terminal glycine deleted-elastin peptide that retains its binding-activity to elastin receptor and inhibits the in vitro and in vivo activities of emphysema induced by VGVAPG [29]. In order to analyze the effect of the PGAIP peptide on the VGVAPG-induced T cell response, activated CD4^+^ T-cells and activated CD8^+^ T-cells were pre-treated 15 min with the peptide PGAIP (10 µg/mL, Genepep SA, Saint Jean de Védas, France) before incubation with the VGVAPG peptide to evaluate the capacity of this peptide to neutralize the VGVAPG peptide.

### Flow cytometry analysis

Analysis of the surface antigens and intracellular cytokines expressed on stimulated CD4^+^ or CD8^+^ sorted T lymphocytes isolated from peripheral blood were performed using antibodies listed in Table 1 and at a target concentration determined from dose/effect specific curves. Four hours before staining, cells were treated with Protein Transport inhibitor according to the manufacturer’s instructions (BD Biosciences). Cells were then treated with Cytofix/CytoPerm (BD Biosciences) before incubation with intracytoplasmic antibodies. Incubation with antibodies was performed during 30 min at + 4 °C, in the dark. Cells were then centrifuged (300g, 10 min, 2 times), and pellets of stained cells were resuspended in 200 µL PBS and stored in the dark at + 4 °C until analysis. Fluorescence emission was assessed by flow cytometry using BD LSRFortessa cell analyzer (BD Biosciences) and BD Diva softwares (BD Biosciences). As compensation controls, BD CompBeads (BD Biosciences) were used and isotype controls of each monoclonal antibody and fluorescence minus one (FMO) tubes were used to assess negative population. A total of 20,000 CD4^+^ T cell events or 20,000 CD8^+^ T cell events were acquired per sample.

**Table 1:** Flow cytometry antibodies

Marker	Fluorochrome	Clone	Provider	Cell target
CD2	APC-H7	RPA-2.10	BD Biosciences	T lymphocytes/NK cells
CD4	PE-Cy™7	SK3	BD Biosciences	Th lymphocytes
CD8	BV510	SK1	BD Biosciences	Tc lymphocytes
CD25	FITC	M-A251	BD Biosciences	Activated T cells
IFN-γ	PE	B27	BD Biosciences	Th1/Tc1 lymphocytes
IL-4	BV421	MP4-25D2	BD Biosciences	Th2/Tc2 lymphocytes
IL-17A	AF^®^647	N49-653	BD Biosciences	Th17/Tc17 lymphocytes
Fixable viability stain 700			BD Biosciences	Live cells

### Quantification of cytokine secretion

Assessment of IFN-γ, IL-4 and IL-17A concentrations in the cell culture supernatants of stimulated CD4^+^ and CD8^+^ T cells was performed using commercially available cytometric bead array (CBA) flex set technology (BD Biosciences, France) according to the manufacturer’s instructions. CBA Flex Set capture bead is a single bead population with distinct fluorescence intensity and is coated with a capture antibody specific for a soluble protein. Preparation of samples was done according to the manufacturer's instructions. Briefly, serial dilutions (1/2, v/v) of the standard preparations were prepared whereas culture supernatants were used undiluted. Then, 50 μL of mixed capture beads were added to each sample. After 1 h incubation period at room temperature, 50 μL of PE detection reagent was added and samples were incubated for 2 h at room temperature. Samples were then washed at 200 g for 5 min and 300 μL wash buffer was added. Flow cytometry analysis was performed using BD LSRFortessa cell analyzer and CBA analysis FCAP array software (BD Biosciences). A total of 700 events were acquired per analyte. The theorical limit detection for each cytokine was 0.5 pg/mL, 0.3 pg/mL and 0.95 pg/mL and for IFN-γ, IL-4 and IL-17A, respectively.

### Desmosine quantification

Assessment of desmosine concentrations in sera was performed in triplicate using a commercially available ELISA kit (Cusabio Biotech product, Interchim, Montluçon, France) according to manufacturer’s instructions. The sensitivity of ELISA kit was 0.039 ng/mL.

### Statistical analysis

Because of the absence of preliminary data regarding the effect of elastin peptides on CD4^+^ and CD8^+^ T-cells in human, the research of this pilot study was exploratory with no power calculation to determine the sample size. The lack of normal distribution of the studied population was checked using Shapiro–Wilk normality test and analysis of non-parametric data were described as the mean ± SD. Difference between two groups were evaluated by using the Wilcoxon-Mann–Whitney test. Correlations were assessed by Spearman’s rank correlation coefficients. Difference between three groups (non-smokers, ex-smokers, current smokers) were evaluated by using the non-parametric Kruskal–Wallis test. Data analysis was performed using Prism 5.1 (GraphPad, La Jolla, CA, USA). A p value lower than 0.05 was considered as significant.

## Results

### Clinical features of study populations

The clinical characteristics of the study population are presented in Table 2. As expected, significant differences in dyspnea, chronic bronchitis, airway obstruction, and hyperinflation were observed between COPD patients and controls. COPD patients were older and had a cumulative smoking exposure higher than controls. No significant difference was observed between the two groups for the percentage of current smokers and peripheral eosinophil count.

**Table 2:** Characteristics of the study population

	COPD patients *n* = 29	Controls *n* = 13
Sex, n (male/female)	15/14	4/9
Age (years)	60 ± 10*	44 ± 19
BMI, kg/m^2^	25 ± 5	27 ± 6
Smoking history		
Never smokers	0% (0)*	46% (6)
Ex-smokers	62% (18)	31% (4)
Smokers	38% (11)	23% (3)
Pack-years	44 ± 18*	18 ± 20
Symptoms		
Dyspnea ≥ 2 mMRC	62% (18)*	8% (1)
Chronic bronchitis	34% (10)*	0% (0)
At least one exacerbation in the previous year	76% (22)	0% (0)
Inhaled treatment		
LAMA and/or LABA	55% (16)	0% (0)
ICS + LABA	31% (9)	0% (0)
Pulmonary function tests		
FEV_1_, L	1.33 ± 0.66*	2.91 ± 1.03
FEV_1_, % predicted	48 ± 18*	101 ± 15
FVC, L	2.7 ± 1.02*	4 ± 0.93
FVC, % predicted	78 ± 21*	107 ± 17
FEV_1_/FVC	0.48 ± 11*	0.79 ± 6
TLC, % predicted	125 ± 24*	103 ± 19
RV, % predicted	203 ± 66*	107 ± 39
Spirometric GOLD 1/2/3–4	1/13/15	NA
GOLD A/B/C/D	6/8/3/12	NA
CT emphysema score (total score/20)^a^	7 ± 5	NA

Values are mean ± standard deviation or percentage (number)

*BMI* body mass index, *LABA* long-acting beat-agonist, *LAMA* long-acting muscarinic antagonist, *ICS* inhaled corticosteroid, *FEV*_*1*_, forced expiratory volume in one second, *FVC* forced vital capacity, *TLC* total lung capacity, *RV* residual volume

*p < 0.05 *vs* control group

^a^Emphysema score was determined using a visual score assigned to each lobe, based on the extent of tissue destruction from 0 (no emphysema) to 4 (> 75% destruction)

### Type 1 (IFN-γ), type 2 (IL-4) and type 17 (IL-17A) cytokine expression

**Fig. 1:**
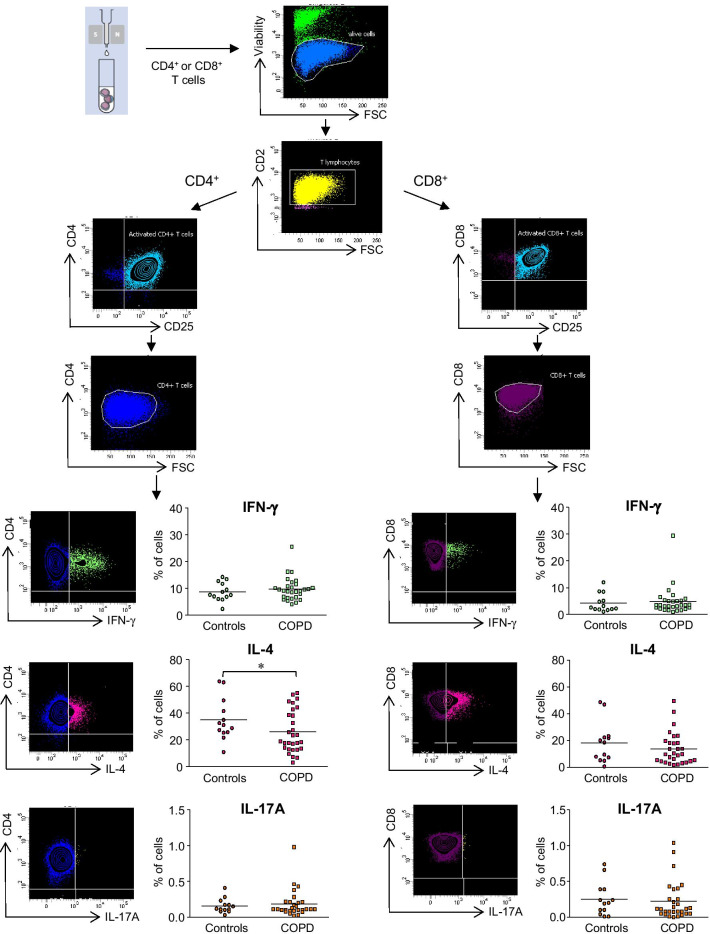
Intracellular cytokine expression by T lymphocytes isolated from COPD patients and controls. Flow cytometry analysis of intracellular cytokine expression (IFN-γ, IL-4 and IL-17A) was performed after anti-CD3/CD28 stimulation of CD4^+^ T-cells or CD8^+^ T-cells sorted from PBMC isolated from COPD patients (n = 29) and controls (n = 13). The plots show sequentially the gating hierarchy of one representative sample: live cells, CD2^+^ T-cells and subsequently activated CD25^+^ CD8^+^ T-cells or activated CD25^+^ CD4^+^ T-cells. The plots on the lowest rows include the cytokine expression gates for either CD8^+^ T-cells or CD4^+^ T-cells. Fluorescence minus one control condition, in which the antibody conjugate in question is omitted, is used to guide creation of the gate that defines positive expression of that target. A total of 20,000 CD4^+^ T-cell events or 20,000 CD8^+^ T-cell events were acquired per sample. Values are expressed as percentage of activated CD4^+^ CD25^+^ or CD8^+^ CD25^+^ T cells expressing each cytokine. *p < 0.05

**Fig. 2:**
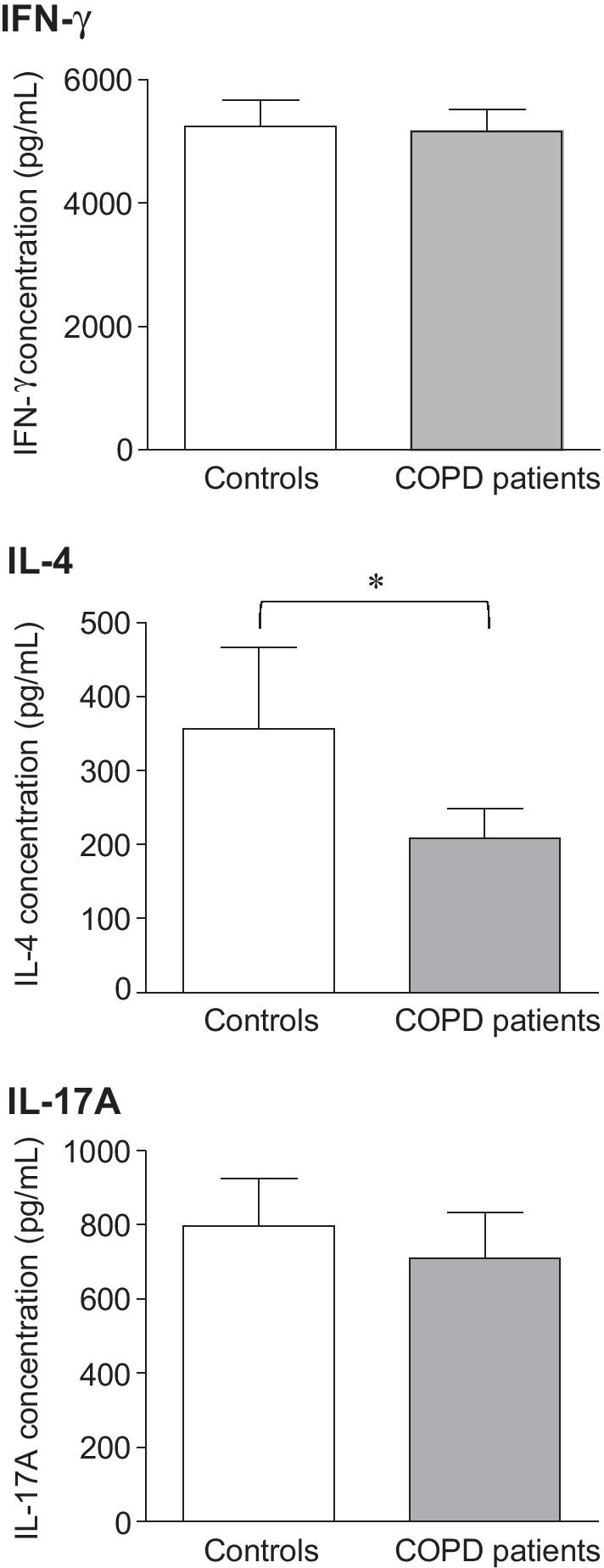
Cytokine production by CD4^+^ T-cells isolated from COPD patients and controls. Quantification of IFN-γ, IL-4 and IL-17A production in culture supernatants of anti-CD3/CD28-stimulated CD4^+^ T-cells isolated from COPD patients (n = 29) and controls (n = 13) was performed by Cytometric Bead Array Analysis. Data are presented as mean ± SD. *p < 0.05

The levels of IFN-γ, IL-4 and IL-17A cytokines expressed by CD4^+^ and CD8^+^ T lymphocytes were analyzed in all COPD patients and controls (Fig. 1). Flow cytometry analysis of anti-CD3/CD28-activated CD4^+^CD25^+^ T-cells showed that the proportion of CD4^+^CD25^+^ T-cells expressing intracellular IL-4 was significantly lower in COPD patients than in controls (26.3 ± 2.4% *vs* 35.3 ± 3.4% respectively, *p* < 0.05), whereas no significant difference was observed in CD4^+^ T cells-derived intracellular IFN-γ (8.5 ± 0.8% in controls *vs* 9.2 ± 2.4% in COPD patients) and IL-17A (0.15 ± 0.07% in controls *vs* 0.18 ± 0.1% in COPD patients) expression (Fig. 1). We also found that the expression of the type-2 specific transcription factor GATA-3 by activated CD4^+^CD25^+^ T-cells was lower in COPD patients compared with control (data not shown). On the other hand, the ability of anti-CD3/CD28-activated CD8^+^CD25^+^ T-cells from COPD patient group to express IFN-γ, IL-4 or IL-17A was preserved, with levels close to those observed in the control group (Fig. 1). Consistent with intracellular analysis of activated CD4^+^CD25^+^ T-cells, IL-4 quantification was significantly decreased in stimulated CD4^+^ T-cells isolated from COPD patients (*p* < 0.05), whereas no difference was observed in CD4^+^ T cell-derived IFN-γ and IL-17A between the different groups (Fig. 2).

### Relationships between IL-4-expression, key features of COPD and levels of desmosine

**Fig. 3:**
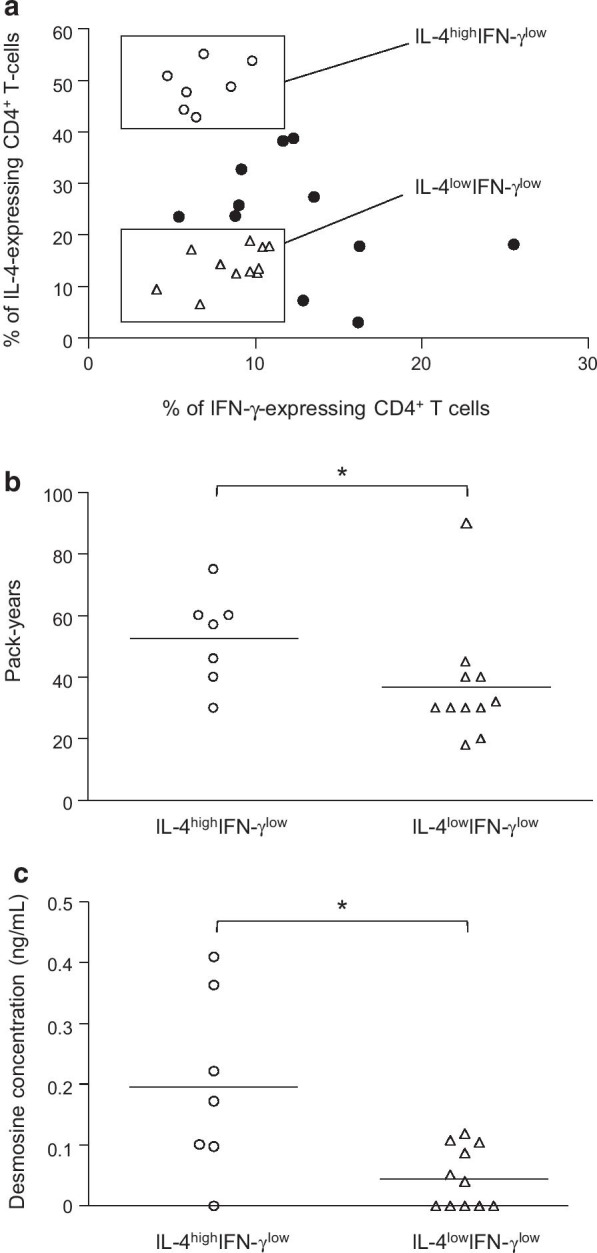
Relationships between IL-4-expression, smoking history and pulmonary elastin degradation. **a** Distribution analysis of CD4^+^ T-cells expressing IFN-γ versus CD4^+^ T-cells expressing IL-4. IL-4^high^IFN-γ^low^: COPD patients (n = 7) with more than 40% of CD4^+^ T-cells expressing IL-4. IL-4^low^IFN-γ^low^: COPD patients (n = 11) with less of 20% of CD4^+^ T-cells expressing IL-4. **b** Relationship between IL-4^high^IFN-γ^low^ and IL-4^low^IFN-γ^low^ subpopulations and exposure to tobacco smoke expressed as number of cigarettes per day for 1 year (pack-years). **c** Relationship between IL-4^high^IFN-γ^low^ and IL-4^low^IFN-γ^low^ subpopulations and concentration of desmosine in the sera of COPD patients. *p < 0.05

We next examined the association between the percentage of IL-4-expressing Th2 cells, and demographic characteristics and key features of COPD including age, sex, tobacco exposure (pack-years, ex-versus current smokers), respiratory symptoms (dyspnea, chronic bronchitis), severity of airway obstruction (FEV_1_ and FEV_1_/FVC) and hyperinflation (residual volume, total lung capacity), CT-scan emphysema score, systemic eosinophils count, history of exacerbation and use of inhaled corticosteroids. In the whole COPD patient series, none of these parameters correlated with the level of IL-4 expression by CD4^+^ T-cells (Table 3). More particularly, we have checked that the mean age difference between the control and COPD groups doesn’t impact on the difference of CD4^+^ T-cells IL-4 expression levels observed between the COPD and control groups. To deeper investigate whether smoking exposure affected IL-4 in COPD, we analyzed the percentage of IL-4-expressing Th2 cells in subgroups of COPD with either high or low level of IL-4 but all with a low concentration of IFN-γ (IL-4^high^IFN-γ^low^ *n* = 7, and IL-4^low^IFN-γ^low^ *n* = 11) (Fig. 3a). The cumulative smoking exposure was significantly higher in the IL-4^high^IFN-γ^low^ than in the IL-4^low^IFN-γ^low^ subgroup (mean pack year of 52.6 ± 15% *vs* 36.8 ± 19%, respectively) (Fig. 3b). We then investigated the association between serum desmosine secretion that reflect pulmonary elastin breakdown in COPD patients, and IL-4 expression. Desmosine concentration in the sera of COPD patients was significantly higher in the IL-4^high^IFN-γ^low^ group than in the IL-4^low^IFN-γ^low^ group (Fig. 3c).

**Table 3:** Correlation between percentage of IL-4-expressing Th2 cells, and demographic characteristics and key features of COPD

	Intracellular IL-4	Secreted IL-4
Age (years)^a^	*p* = 0.57	*p* = 0.49
	*r* = − 0.11	r = 0.14
Sex (male/female)^b^	*p* = 0.42	p = 0.68
Smoking (pack years)^a^	p = 0.95	r = 0.32
	r = 0.01	r = 0.20
Smoking (ex-smokers *vs* current-smokers)^b^	*p* = 0.70	*p* = 0.06
Chronic bronchitis (yes/no)^b^	*p* = 0.25	*p* = 0.16
Dyspnea scale (mMRC)^a^	*p* = 0.83	*p* = 0.18
	*r* = 0.04	r = − 0.26
FEV_1_ (%)^a^	*p* = 0.66	*p* = 0.19
	*r* = − 0.08	*r* = − 0.25
FVC (%)^a^	*p* = 0.76	*p* = 0.34
	r = 0.06	*r* = − 0.19
FEV_1_/FVC (%)^a^	*p* = 0.25	*p* = 0.51
	*r* = − 0.21	*r* = − 0.13
RV (%)^a^	*p* = 0.07	*p* = 0.71
	*r* = 0.32	*r* = 0.07
TCL (%)^a^	*p* = 0.11	*p* = 0.53
	*r* = 0.30	*r* = 0.02
RV/TCL (%)^a^	*p* = 0.56	*p* = 0.49
	*r* = 0.11	*r* = 0.02
Emphysema (yes/no)^b^	*p* = 0.44	*p* = 0.88
Emphysema score^a^	*p* = 0.40	*p* = 0.48
	*r* = 0.16	*r* = 0.14
Eosinophils (%)^a^	*p* = 0.14	*p* = 0.17
	*r* = − 0.28	*r* = 0.26
Eosinophils (total count)^a^	*p* = 0.52	*p* = 0.71
	*r* = − 0.12	*r* = 0.07
Exacerbations (n per year)^a^	*p* = 0.82	*p* = 0.46
	r = 0.04	*r* = − 0.15
ICS (yes/no)^b^	*p* = 0.27	*p* = 0.50

*mMRC* modified Medical Research Council, *FEV*_*1*_ forced expiratory volume in one second, *FVC* forced vital capacity, *RV* residual volume, *ICS* inhaled corticosteroid

^a^Quantitative data were evaluated by Spearman correlation test (*p* and *r* values)

^b^Qualitative data were evaluated by Mann and Whitney test (*p* values only)

### Elastin peptide (EP) affect IL-4 expression in COPD patients

**Fig. 4:**
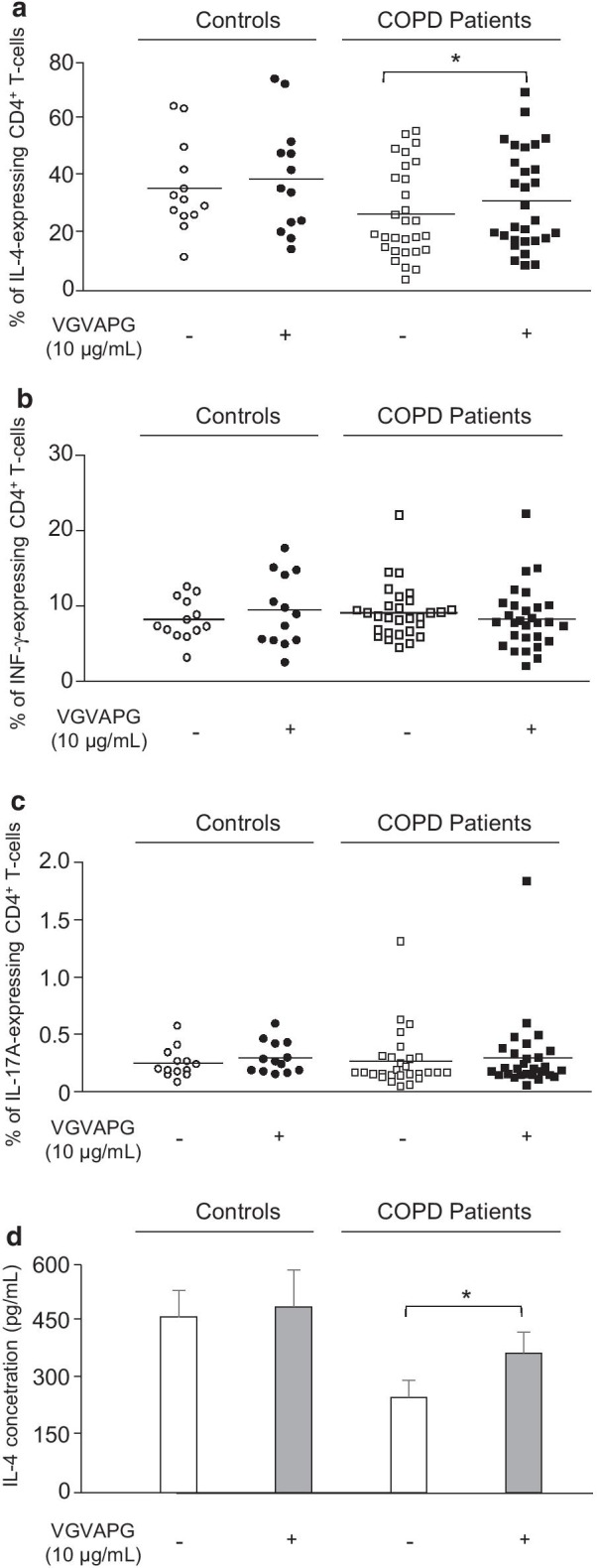
The VGVAPG elastin peptide affect IL-4 expression in Th2 cells from COPD patients. CD4^+^ T-cells isolated from COPD patients (n = 29) and controls (13) were stimulated with anti-CD3/CD28 beads in the presence or not of the VGVAPG peptide (10 µg/mL). After 48 h incubation, stimulated cells were collected, stained and analyzed by flow cytometry to evaluate the levels of **a** IL-4, **b** IFN-γ and **c** IL-17A intracellular cytokine expression. *p < 0.05. **d** Quantification of IL-4 production in culture supernatants of anti-CD3/CD28-stimulated CD4^+^ T-cells in the presence or not of the VGVAPG peptide (10 µg/mL). Quantification was performed by Cytometric Bead Array Analysis. Data are presented as mean ± SD. *p < 0.05

Desmosine is a specific marker of elastin peptide (EP) genesis and EP modulate the expression of pro-inflammatory cytokines in human monocytes and lymphocytes [26, 27]. We then investigated whether EP, i.e. the bioactive VGVAPG elastin peptide, modulate cytokine production in CD4^+^ and CD8^+^ T cells isolated from COPD patients and controls. Treatment of CD4^+^ T-cells from COPD patients with 10 µg of the VGVAPG peptide significantly increased the proportion of CD4^+^ T-cells expressing intracellular IL-4 (Fig. 4a). No significant effect of the VGVAPG peptide on IL-4 expression was shown in CD4^+^ T-cells from controls (Fig. 4a). The VGVAPG peptide did not affect IFN-γ and IL-17A expression in CD4^+^ T-cells from COPD patients (Fig. 4b, c) and did not modify cytokine expression in CD8^+^ T-cells from either controls or COPD patients (data not shown). The VGVAPG peptide effects were also clearly demonstrated by IL-4 protein secretion quantification (Fig. 4d).

### S-gal-specific antagonist peptide inhibits VGVAPG effects on IL4

**Fig. 5:**
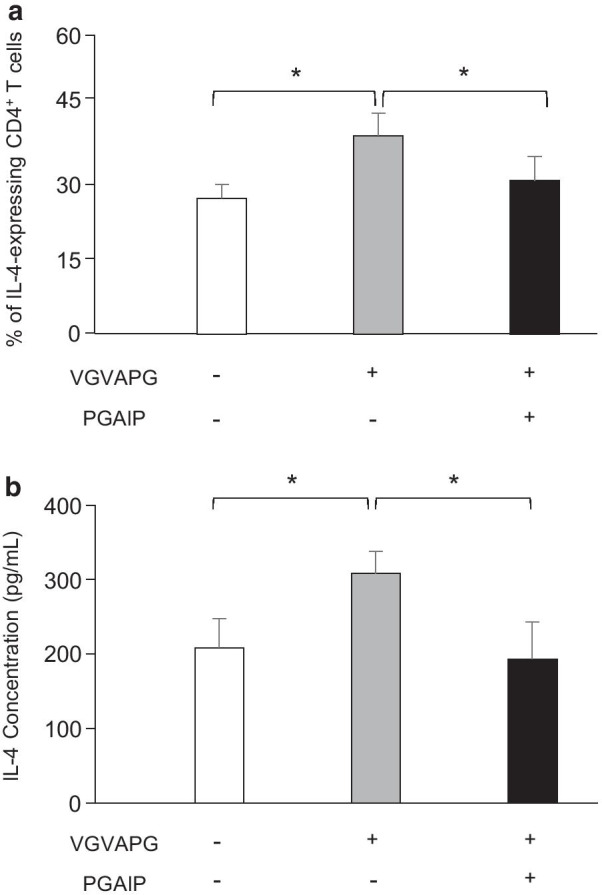
S-gal-specific antagonist peptide inhibits VGVAPG effects on IL4-expressing Th2 cells in COPD patients*.* **a** CD4^+^ T-cells isolated from COPD patients (n = 6) were stimulated with anti-CD3/CD28 beads and pre-treated or not with the analogous antagonist peptide PGAIP (10 µg/mL) 15 min before incubation or not with the VGVAPG peptide (10 µg/mL). After 48 h co-incubation, stimulated cells were collected, stained and analyzed by flow cytometry to evaluate the levels of IL-4 intracellular cytokine expression. *p < 0.05. **b** Quantification of IL-4 production in culture supernatants of anti-CD3/CD28-stimulated CD4^+^ T-cells in the presence or not of the PGAIP (10 µg/mL) and/or VGVAPG peptide (10 µg/mL). Quantification was performed by Cytometric Bead Array Analysis. Data are presented as mean ± SD. *p < 0.05

We next investigated whether the increase of IL-4 expression by CD4^+^ T-cells in response to the VGVAPG peptide treatment was linked to its interaction with the elastin receptor S-gal. To that purpose and before incubation with the VGVAPG peptide, CD4^+^ T cells isolated from COPD patients were pre-treated with the analogous peptide PGAIP that antagonizes the EP/S-gal interactions [29]. In activated CD4^+^ T-cells treated both with the PGAIP and the VGVAPG peptides, the proportion of IL-4-expressing CD4^+^ T-cell was significantly reduced compared to CD4^+^ T-cells in which VGVAPG was used alone and reached values comparable to those obtained in VGVAPG-untreated CD4^+^ T-cells (Fig. 5a). As expected, this negative shift induced by the PGAIP peptide correlates with a significant decreased of the IL-4 protein secretion (Fig. 5b).

## Discussion

The main results of our study indicate that the elastin peptide VGVAPG modulates CD4^+^ T-Cell IL-4 production in patients with COPD. Indeed, the VGVAPG treatment of CD4^+^ T-cells increased the percentage of cells expressing IL-4 in COPD patients, and these effects were inhibited in the presence of analogous peptide antagonizing EP/elastin receptor (S-gal) interactions. Our study also demonstrates a high heterogeneity in the percentage of T cells expressing IL-4 in COPD patients with an interesting association between IL-4 expression and the levels of desmosine, a marker of elastin breakdown. In VGAPG non stimulated cells, the percentage of IL-4 was decreased in COPD patients compared to controls, whereas no significant differences were found between the COPD patients and controls with regard to levels of IFN-γ and IL-17A.

Several other studies have characterized the profile of cytokine production in the peripheral blood of COPD patients, with conflicting results. Some have shown an increased expression of the type 2 cytokine IL-4 by CD4^+^ or CD8^+^ circulating T lymphocytes [13, 30], whereas others found a predominant type 1 and/or type 17 cytokine pattern [15, 16]. These contradictory results could be explained by differences in experimental design and study population. Our findings contrast with those of these reports, but it is noteworthy that a decrease of IL-4 expression by CD4^+^ T-cells in the peripheral blood of COPD patients has been already described [11]. This decrease of IL-4 expression was concomitant with an increase expression of the type 1 cytokine IFN-γ by the CD4^+^ T subset, suggesting a correlation between a low Th2 expression and the development of a Th1 phenotype in patients with COPD [11].

The Th1/Th2 balance that reflects the immunological status in COPD patients can be determined through the IFN-γ/IL-4 ratio analysis. A recent study demonstrated that in COPD patients with acute exacerbation the level of Th1 was reduced whereas the level of Th2 was predominant. Oppositely, Th2 cells have been shown to decrease in stable COPD patients while Th1 cells increased [31]. In our study, using an IFN-γ-expressing CD4^+^ T-cells percentage of < 12% as threshold, we identified and selected two distinct groups of patients: (i) a first group of patients with more than 40% of CD4^+^ T-cells expressing IL-4 (IL-4^high^IFN-γ^low^) and (ii) a second group with less of 20% of CD4^+^ T-cells expressing IL-4 (IL-4^low^IFN-γ^low^). COPD patients belonging to the IL-4^high^IFN-γ^low^ group had a cumulative exposure to tobacco smoke and a desmosine concentration significantly higher than those belonging to the IL-4^low^IFN-γ^low^ group, highlighting the critical role of tobacco exposure in elastin degradation.

A correlation between smoking and enhanced concentrations of EP in biological fluids of COPD patients has been previously described [32, 33]. We here demonstrated that this correlation is also related with increased level of Th2 cytokine (IL-4) expression, and we revealed that CD4^+^ T-cells from COPD patients increased synthesis and release of IL-4 in response to the elastin peptide VGVAPG. The effects of EP on Th2 cytokine expression by CD4^+^ T-cells from COPD patients were mediated via the elastin receptor S-gal, as the S-gal-specific antagonist PGAIP peptide [29] blocked EP-related IL-4 production.

## Conclusions

As a whole, these results highlight (1) the high heterogeneity in the COPD subpopulation in terms of EP genesis and consequently CD4^+^ T-cell dependent IL-4 expression; (2) the interest to evaluate IL-4 in circulating T-cells to better characterize COPD inflammatory phenotypes and to develop further studies to precise the putative role of EP to favour an IL-4-dependent anti-inflammatory response in COPD patients. In a clinical and therapeutic perspective, monitoring and modulating CD4^+^ T-cells IL-4 production via the VGVAPG/S-gal receptor may lead to the development of innovative personalized biological treatments in COPD.

## Data Availability

All data generated or analyzed during this study are included in this published article [and its supplementary information files].

## References

[1] Global strategy for the diagnosis, management, and prevention of chronic obstructive pulmonary disease, Global initiative for Chronic Obstructive Lung Disease (GOLD) 2017 report. www.Goldcopd.org. Accessed Feb 2018.

[2] Barnes PJ (2016). Inflammatory mechanisms in patients with chronic obstructive pulmonary disease. J Allergy Clin Immunol.

[3] Urbanowicz RA, Lamb JR, Todd I, Corne JM, Fairclough LC (2010). Enhanced effector function of cytotoxic cells in the induced sputum of COPD patients. Respir Res.

[4] Freeman CM, Stolberg VR, Crudgington S, Martinez FJ, Han MK, Chensue SW, Arenberg DA, Meldrum CA, McCloskey L, Curtis JL (2014). Human CD56^+^ cytotoxic lung lymphocytes kill autologous lung cells in chronic obstructive pulmonary disease. PLoS ONE.

[5] Grumelli S, Corry DB, Song LZ, Song L, Green L, Huh J, Hacken J, Espada R, Bag R, Lewis DE, Kheradmand F (2004). An immune basis for lung parenchymal destruction in chronic obstructive pulmonary disease and emphysema. PLoS Med.

[6] Harrison OJ, Foley J, Bolognese BJ, Long E, Podolin PL, Walsh PT (2008). Airway infiltration of CD4^+^ CCR6^+^ Th17 type cells associated with chronic cigarette smoke induced airspace enlargement. Immunol Lett.

[7] Saetta M, Di Stefano A, Turato G, Facchini FM, Corbino L, Mapp CE, Maestrelli P, Ciaccia A, Fabbri LM (1998). CD8^+^ T-lymphocytes in peripheral airways of smokers with chronic obstructive pulmonary disease. Am J Respir Crit Care Med.

[8] Di Stefano A, Caramori G, Capelli A, Gnemmi I, Ricciardolo FL, Oates T, Donner CF, Chung KF, Barnes PJ, Adcock IM (2004). STAT4 activation in smokers and patients with chronic obstructive pulmonary disease. Eur Respir J.

[9] Di Stefano A, Caramori G, Gnemmi I, Contoli M, Vicari C, Capelli A, Magno F, D’Anna SE, Zanini A, Brun P, Casolari P, Chung KF, Barnes PJ, Papi A, Adcock I, Balbi B (2009). T helper type 17-related cytokine expression is increased in the bronchial mucosa of stable chronic obstructive pulmonary disease patients. Clin Exp Immunol.

[10] Hogg JC, Chu F, Utokaparch S, Woods R, Elliott WM, Buzatu L, Cherniak RM, Rogers RM, Sciurba FC, Coxson HO, Paré PD (2004). The nature of small-airway obstruction in chronic obstructive pulmonary disease. N Engl J Med.

[11] Majori M, Corradi M, Caminati A, Cacciani G, Bertacco S, Pesci A (1999). Predominant Th1 cytokine pattern in peripheral blood from subjects with chronic obstructive pulmonary disease. J Allergy Clin Immunol.

[12] Zhang MQ, Wan Y, Jin Y, Xin JB, Zhang JC, Xiong XZ, Chen L, Chen G (2014). Cigarette smoking promotes inflammation in patients with COPD by affecting the polarization and survival of Th/Tregs through up-regulation of muscarinic receptor 3 and 5 expression. PLoS ONE.

[13] Mattoli S, Kleimberg J, Stacey MA, Bellini A, Sun G, Marini M (1997). The role of CD8^+^ Th2 lymphocytes in the development of smoking related lung damage. Biochem Biophys Res Comm.

[14] Makris D, Lazarou S, Alexandrakis M, Kourelis TV, Tzanakis N, Kyriakou D, Gourgoulianis KI (2008). Tc2 response at the onset of COPD exacerbations. Chest.

[15] Vargas-Rojas MI, Ramirez-Venegas A, Limón-Camacho L, Ochoa L, Hernández-Zenteno R, Sansores RH (2011). Increase of Th17 cells in peripheral blood of patients with chronic obstructive pulmonary disease. Respir Med.

[16] Chang Y, Nadigel J, Boulais N, Bourbeau J, Maltais F, Eidelman DH, Hamid Q (2011). CD8 positive T cells express IL-17 in patients with chronic obstructive pulmonary disease. Respir Res.

[17] Sinden NJ, Baker MJ, Smith DJ, Kreft JU, Dafforn TR, Stockley RA (2015). α-1-antitrypsin variants and the proteinase/antiproteinase imbalance in chronic obstructive pulmonary disease. Am J Physiol Lung Cell Mol Physiol.

[18] Lucey EC, Keane J, Kuang PP, Snider GL, Goldstein RH (2002). Severity of elastase-induced emphysema is decreased in tumor necrosis factor-alpha and interleukin-1beta receptor-deficient mice. Lab Invest.

[19] Houghton AM, Quintero PA, Perkins DL, Kobayashi DK, Kelley DG, Marconcini LA, Mecham RP, Senior RM, Shapito SD (2006). Elastin fragments drive disease progression in a murine model of emphysema. J Clin Invest.

[20] Eurlings IM, Dentener MA, Mercken EM, de Cabo R, Bracke KR, Vernooy JH, Wouters EF, Reynaert NL (2014). A comparative study of matrix remodeling in chronic models for COPD; mechanistic insights into the role of TNF-α. Am J Physiol Lung Cell Mol Physiol.

[21] Lombard C, Arzel L, Bouchu D, Wallach J, Saulnier J (2006). Human leukocyte elastase hydrolysis of peptides derived from human elastin exon 24. Biochimie.

[22] McClintock DE, Starcher B, Eisner MD, Thompson BT, Hayden DL, Church GD, Matthay MA (2006). Higher urine desmosine levels are associated with mortality in patients with acute lung injury. Am J Physiol Lung Cell Mol Physiol.

[23] Betsuyaku T, Nishimura M, Yoshioka A, Takeyabu K, Miyamoto K, Kawakami Y (1996). Elastin-derived peptides and neutrophil elastase in bronchoalveolar lavage fluid. Am J Respir Crit Care Med.

[24] Dupont A, Dury S, Gafa V, Lebargy F, Deslée G, Guenounou M, Antonicelli F, Le Naour R (2013). Impairment of neutrophil reactivity to elastin peptides in COPD. Thorax.

[25] Hinek A, Rabinovitch M, Keeley F, Okamura-Oho Y, Callahan J (1993). The 67-kD elastin/laminin-binding protein is related to an enzymatically inactive, alternatively spliced form of beta-galactosidase. J Clin Invest.

[26] Baranek T, Debret R, Antonicelli F, Lamkhioued B, Belaaouaj A, Hornebeck W, Bernard P, Guenounou M, Le Naour R (2007). Elastin receptor (spliced galactosidase) occupancy by elastin peptides counteracts pro-inflammatory cytokine expression in lipopolysaccharide-stimulated human monocytes through NF-kappaB down-regulation. J Immunol.

[27] Debret R, Antonicelli F, Theill A, Hornebeck W, Bernard P, Guenounou M, Le Naour R (2005). Elastin-derived peptides induce a T-helper type 1 polarization of human blood lymphocytes. Arterioscler Thromb Vasc Biol.

[28] Meghraoui-Kheddar A, Pierre A, Sellami M, Audonnet S, Lemaire F, Le Naour R (2013). Elastin receptor (S-gal) occupancy by elstin peptides modulates T-cell response during murine emphysema. Am J Physiol Lung Cell Mol Physiol.

[29] Sellami M, Meghraoui-Kheddar A, Terryn C, Fichel C, Bouland N, Diebold MD, Guenounou M, Héry-Huynh S, Le Naour R (2016). Induction and regulation of murine emphysema by elastin peptides. Am J Physiol Lung Cell Mol Physiol.

[30] Solleiro-Villavicencio H, Quintana-Carillo R, Falfán-Valencia R, Vargas-Rojas MI (2015). Chronic obstructive pulmonary disease induced by exposure to biomass smoke is associated with Th2 cytokine production profile. Clin Immunol.

[31] Sun J, Liu T, Yan Y, Huo K, Zhang W, Liu H, Shi Z (2018). The role of Th1/Th2 cytokines played in regulation of specific CD4^+^ Th1 cell conversion and activation during inflammatory reaction of chronic obstructive pulmonary disease. Scand J immunol.

[32] Gottlieb DJ, Stone PJ, Sparrow D, Gale ME, Weiss ST, Snider GL, O’Conner GT (1996). Urinary desmosine excretion in smokers with and without rapid decline of lung function: the Normative Aging Study. Am J Respir Crit Care Med.

[33] Schriver EE, Davidson JM, Sutcliffe MC, Swindell BB, Bernard GR (1992). Comparison of elastin peptide concentrations in body fluids from healthy volunteers, smokers, and patients with chronic obstructive pulmonary disease. Am Rev Respir Dis.

